# Integrative conjugative elements of the ICE*Pan* family play a potential role in *Pantoea ananatis* ecological diversification and antibiosis

**DOI:** 10.3389/fmicb.2015.00576

**Published:** 2015-06-08

**Authors:** Pieter De Maayer, Wai-Yin Chan, Douglas A. J. Martin, Jochen Blom, Stephanus N. Venter, Brion Duffy, Don A. Cowan, Theo H. M. Smits, Teresa A. Coutinho

**Affiliations:** ^1^Centre for Microbial Ecology and Genomics, University of PretoriaPretoria, South Africa; ^2^Department of Microbiology, University of PretoriaPretoria, South Africa; ^3^Forestry and Agricultural Biotechnology Institute, University of PretoriaPretoria, South Africa; ^4^Bioinformatics and Systems Biology, Justus Liebig University GiessenGiessen, Germany; ^5^Environmental Genomics and Systems Biology Research Group, Institute of Natural Resource Sciences, Zürich University of Applied SciencesWädenswil, Switzerland; ^6^Department of Genetics, University of PretoriaPretoria, South Africa

**Keywords:** *Pantoea ananatis*, integrative and conjugative element, ICE*Pan*, stress response, antibiosis, *umuDC*

## Abstract

*Pantoea ananatis* is a highly versatile enterobacterium isolated from diverse environmental sources. The ecological diversity of this species may be attributed, in part, to the acquisition of mobile genetic elements. One such element is an Integrative and Conjugative Element (ICE). By means of *in silico* analyses the ICE elements belonging to a novel family, ICE*Pan*, were identified in the genome sequences of five *P. ananatis* strains and characterized. PCR screening showed that ICE*Pan* is prevalent among *P. ananatis* strains isolated from different environmental sources and geographic locations. Members of the ICE*Pan* family share a common origin with ICEs of other enterobacteria, as well as conjugative plasmids of *Erwinia* spp. Aside from core modules for ICE*Pan* integration, maintenance and dissemination, the ICE*Pan* contain extensive non-conserved islands coding for proteins that may contribute toward various phenotypes such as stress response and antibiosis, and the highly diverse ICE*Pan* thus plays a major role in the diversification of *P. ananatis*. An island is furthermore integrated within an ICE*Pan* DNA repair-encoding locus *umuDC* and we postulate its role in stress-induced dissemination and/or expression of the genes on this island.

## Introduction

*Pantoea ananatis* is a ubiquitous and versatile enterobacterial species, with strains isolated globally from a wide range of environmental sources. Most commonly isolated from plants, *P. ananatis* has been identified as the causative agent of diseases on a wide range of host plants, including agronomically important crops such as rice, corn, onion, and *Eucalyptus* (Coutinho and Venter, 2009). Other isolates represent non-pathogenic endo- or epiphytes, while a plant growth promoting *P. ananatis* strain has recently been characterized and patented (Coutinho and Venter, 2009; Kim et al., 2012). The potential of *P. ananatis* as an effective biological control agent of a number of phytopathogenic bacteria and fungi is also being investigated (Gasser et al., 2012). Furthermore, *P. ananatis* has been associated with human disease (De Baere et al., 2004). The wide ecological distribution and versatile lifestyles of *P. ananatis* suggests that this bacterial species has undergone extensive genetic adaptation in order to effectively occupy and exploit its various ecological niches.

A key driver of rapid bacterial adaptation is the acquisition of genetic material through the horizontal exchange of mobile genetic elements. These elements, which include plasmids, phages, transposons and Integrative and Conjugative Elements (ICEs), influence bacterial fitness and allow microorganisms to occupy novel niches (Wozniak and Waldor, 2010; Aminov, 2011). ICE elements are a class of self-transmissible integrative elements found in numerous Gram-positive and Gram-negative bacterial taxa (Bi et al., 2012). At present, only a limited number of ICEs have been classified into ICE families, including the well-known SXT/R391 family found in *Vibrio* and *Providencia* spp. (Wozniak and Waldor, 2010). With the exponential increase of available genome sequences, it can be envisaged that many novel integrative and conjugative elements will be identified and novel ICE families described.

Typically, ICEs comprise of three core modules required for functioning of the element. The first module includes an integrase gene (*xerC*) which ensures the site-specific chromosomal integration of the ICE as well as effective excision of the element, where it may be aided by an excisionase or recombination directionality factor (Burrus et al., 2006; Wozniak and Waldor, 2010). Following excision, the ICE forms a circular extrachromosomal element through recombination between identical sequences at both ends (Burrus et al., 2006). The second core module is involved in the conjugative transfer of the circularized ICE. This module generally comprises a Type IV secretion system (T4SS), which ensures intimate contact between the donor and recipient for dissemination of the ICE (Wozniak and Waldor, 2010). The third core module is involved in ICE maintenance and includes regulatory proteins as well as toxin–antitoxin and partition systems which ensure that the ICE is vertically transmitted within a bacterial lineage (Wozniak and Waldor, 2009). Aside from core modules, ICEs contain an extensive array of cargo genes, which may contribute to diverse phenotypes (Wozniak and Waldor, 2010). These include genes coding for factors involved in pathogenesis, metabolic adaptation, the production of secondary metabolites and resistance to antibiotics and heavy metals (Burrus et al., 2002). Homologous recombination between ICEs may also occur in ICE recipients, leading to the formation of hybrid ICEs, which contribute to the diversity of ICE elements and potential accessory factors they encode (Wozniak and Waldor, 2010).

Previous comparative genomic analyses (De Maayer et al., 2014) revealed the presence of a large genomic island in five of eight compared *P. ananatis* genomes. Here we have characterized these genomic islands and show that they represent ICE elements, which are prevalent among strains of the species. The ICE*Pan* elements comprise core modules interspersed with divergent cargo regions. Proteins encoded in these cargo regions may contribute to stress-response and production of antibiotic secondary metabolites. Finally, we identified a non-conserved island situated within the *umuC* gene involved in DNA damage repair and postulate its potential role in the dissemination or expression of the cargo genes.

## Methods

### *In silico* characterization of the ICE*Pan* elements

ICE elements were identified on the genomes of five *P. ananatis* strains (Table 1) by localized tBlastN analysis with protein coding sequences from known ICE element core modules using BioEdit v 7.1.11 (Hall, 1999). The complete genome sequences of *P. ananatis* AJ13355 (NC_017531.1), LMG5342 (NC_016816.1) and PA13 (CP003085.1), as well as the draft genomes of *P. ananati*s B1-9 (CAEI00000000) and BD442 (JMJL00000000) are publically available on the NCBI database under the given accession numbers. The full extent of the ICE*Pan* elements was elucidated by BlastN with the full tRNA-Phe nucleotide sequence of the ICE-negative *P. ananatis* LMG20103 (PANA_t0061) (De Maayer et al., 2010) to determine the ICE*Pan* integration site. The sizes and G+C contents of the ICE*Pan* Island (IR) and Core (CR) regions and other sequence manipulations, such as sequence alignments and localized Blast comparisons were performed using BioEdit (Hall, 1999). The CDS sets encoded on the ICE*Pan* elements were standardized using the FgenesB ORF prediction server (http://www.softberry.com) and reciprocal best hit (RBH) BlastP analysis (Altschul et al., 1990; Moreno-Hagelsieb and Latimer, 2008). Core module CDSs in ICE*Pan* elements were predicted by localized BlastP of the protein products with orthologs from the ICEberg server (Bi et al., 2012; http://db-mml.sjtu.edu.cn/ICEberg/). The core module CDSs were further used to identify ICE-like elements in closely related organisms by BlastP comparison against the NCBI non-redundant protein sequences (nr) database. Average amino acid identities were calculated on the basis of localized RBH BlastP analyses (Moreno-Hagelsieb and Latimer, 2008), where the sum of the number of identities was divided by the sum of the total lengths of the aligned regions. Orthology of the CDSs encoded on the ICE*Pan* IR regions to proteins encoded on other genomes was determined by BlastP comparison of the translated protein products against the NCBI nr database, while conserved domains were predicted using the Batch CD-search tool and the Conserved Domain Database (Marchler-Bauer and Bryant, 2004; Marchler-Bauer et al., 2009).

**Table 1:** **General characteristics of ICE***Pan***-carrying ***P. ananatis*** strains and the ICE***Pan*** elements**.

**Strain**	**Host**	**Lifestyle**	**Integration site**	**Size (kb)**	**G+C (%)**	**# CDSs**	**References**
AJ13355	Soil	Saprophyte	2	59.4	53.22	59	Hara et al., 2011
B1-9	Onion	Plant-growth promoter	1	99.2	53.73	110	Kim et al., 2012
BD442	Corn	Pathogen	1	98.8	53.21	107	Weller-Stuart et al., 2014
PA13	Rice	Pathogen	1	107.2	53.87	108	Choi et al., 2012
LMG5342	Human	Pathogen	1	110.3	53.25	113	De Maayer et al., 2012

*The P. ananatis strain names, their hosts and lifestyles are shown. The size of the complete ICEPan element for each strain, G+C contents and number of CDSs encoded on the elements are indicated. The integration site of the ICEPan elements in each strain, (Site 1, adjacent to yjdC encoding a putative HTH-transcriptional regulator; Site 2, adjacent to serine phosphatase gene rbsU) are indicated*.

### Phylogenetic tree construction

Phylogenetic trees were constructed, using the amino acid sequences of the chromosomal house-keeping markers AtpD, GyrB, InfB, and RpoB; ICE*Pan* core CDSs and enterobacterial ICE core CDSs (ICE*Epir*CFBP5888, ICE*Ecl*ATCC13047, YAPI, Ctnscr94, and ICE*Pwa*WPP163); ICE*Pan* core CDSs and conjugative plasmid core CDSs (pEI70 and pEb102) and *umuDC* island CDSs. The amino acid sequences were concatenated and aligned using the MAFFT web server (Katoh and Standley, 2013). Tree construction was performed using MEGA v 5.2 (Tamura et al., 2011) with the Neighbor-joining approach, with complete gap deletion, Poisson correction and bootstrapping (*n* = 1000).

### PCR screening for ICE*Pan* elements in *P. ananatis* strains

Forty-six *P. ananatis* strains of different geographic origins and sources of isolation (Table 2) were incubated overnight at 28°C and maintained on Luria Bertani (LB) agar. Genomic DNA was extracted using the ZR Fungal/Bacterial DNA Microprep™ kit (Zymo Research Corporation, California, USA), as per the manufacturer's instructions. Strains were confirmed as belonging to the species *P. ananatis* by PCR amplification and Sanger sequencing of the 16S rRNA gene. The chromosomal DNAs were PCR amplified using the following primer sets: *sit1F*-*sit1R* and *sit2F-sit2R*, covering the *hyp*-tRNA-Phe-*yjdC* region and *hyp*-tRNA-Phe-*rbsU* regions of ICE*Pan*-negative *P. ananatis* strains for which genome sequences are available, respectively; *xerC1F*-*xerC1R* and *xerC2F-xerC2R*, within the XerC1 and XerC2 integrases of site 1 and site 2-integrated ICE*Pan* elements, respectively; *topBF-topBR, pilVF-pilVR*, and *traIF-traIR*, designed on the nucleotide sequences of *topB, pilV*, and *traI* core module genes, respectively; *mae1F-mae1R* and *ssfDF-ssfDR*, designed from the nucleotide sequences of *ssfD* in IR-4A and *mae1* in IR-4B, respectively (Table S1).

**Table 2:** **The presence or absence of ICE***Pan*** in ***P. ananatis*** strain isolated from different ecological sources**.

**Strain Acc**.	**Origin**	**Host**	***sit1***	***sit2***	***xerC1***	***xerC2***	***topB***	***pilV***	***traI***	***ssfD***	***mae1***
FBCC0024	RSA	Insect	+	−	−	−	−	−	−	−	−
**PA13**	**Korea**	**Rice**	+	−	+	−	+	+	+	+	+
**B1−9**	Korea	Tea	+	−	+	−	+	+	+	+	+
FBCC0053	RSA	Insect	+	−	+	−	+	+	+	−	−
FBCC0083	USA	Onion	+	−	+	−	+	+	+	−	−
**BD442**	RSA	Corn	+	−	+	−	+	+	+	−	−
**LMG5342**	USA	Human	+	−	+	−	+	+	+	−	+
LMG20105	RSA	*Eucalyptus*	+	−	−	−	+	+	+	+	−
FBCC0030	RSA	Insect	+	−	+	+	+	+	+	−	+
LMG2678	Zimbabwe	Wheat	+	−	−	+	+	+	+	−	−
FBCC0094	USA	Onion	+	−	−	−	+	+	+	−	−
0197−28	USA	Sudangrass	+	−	−	−	+	+	+	−	−
0696−21	USA	Sudangrass	+	−	−	−	+	−	+	−	−
FBCC0087	USA	Onion	+	−	+	−	+	−	−	−	−
ICMP12183	Brazil	Cassia	+	−	−	−	+	−	+	−	−
BD301	RSA	Onion	+	−	−	−	−	+	−	−	−
LMG2101	India	Rice	+	−	−	−	−	−	−	−	−
BD377	RSA	Onion	+	−	−	−	−	−	−	−	−
**PA4**	RSA	Onion	+	−	−	−	−	−	−	−	−
DAR76141	Aus	Rice	−	+	+	−	+	+	+	+	−
DAR76144	Aus	Rice	−	+	+	+	+	+	+	−	−
RAMI7969	Aus	Rice	−	+	−	+	+	+	+	+	−
FBCC0583	Uruguay	*Eucalyptus*	−	+	−	−	+	+	+	−	−
LMG2628	Japan	Banana	−	+	−	+	−	+	+	−	−
Yomagi−101	Japan	*Artemisia* sp.	−	+	−	−	+	+	+	−	+
**AJ13355**	Japan	Soil	−	+	−	+	−	−	+	+	+
B731	Brazil	Corn	−	+	−	+	−	+	−	−	+
LMG2676	USA	Wheat	−	+	−	+	−	+	+	−	−
ATCC35400	USA	Melons	−	+	−	−	−	−	+	−	−
LMG2675	Europe	Wheat	−	+	−	−	−	−	−	−	−
BD588	RSA	Corn	−	+	−	−	−	−	−	−	−
SUPP2582	Japan	Melon	−	−	−	−	+	+	+	−	+
BD561	RSA	Corn	−	−	−	−	−	+	−	−	−
**LMG2665^T^**	**USA**	**Pineapple**	−	−	−	−	−	−	−	−	−
FBCC0116	RSA	*Eucalyptus*	−	−	−	−	−	−	−	−	−
**LMG20103**	RSA	*Eucalyptus*	−	−	−	−	−	−	−	−	−
LMG2666	USA	Pineapple	−	−	−	−	−	−	−	−	−
SUPP2219	Japan	Rice	−	−	−	−	−	−	−	−	−
LMG2807	USA	*Cattleya* sp.	−	−	−	−	−	−	−	−	−
BD622	RSA	Corn	−	−	−	−	−	−	−	−	−
FBCC0367	Thailand	*Eucalyptus*	−	−	−	−	−	−	−	−	−
SUPP1128	Japan	Melon	−	−	−	−	−	−	−	−	−
SUPP1791	Japan	Melon	−	−	−	−	−	−	−	−	−
SUPP2213	Japan	Rice	−	−	−	−	−	−	−	−	−
SUPP2219	Japan	Rice	−	−	−	−	−	−	−	−	−
ICMP10132	Brazil	Sugarcane	−	−	−	−	−	−	−	−	−

*P. ananatis strains were obtained from various commercial and research culture collections: FBCC accessions – Forestry and Agricultural Biotechnology Institute Bacterial Culture Collection, T.A. Coutinho, University of Pretoria, South Africa; LMG accessions – BCCM/LMG culture collection, University of Gent, Belgium; BD and PA accessions – Plant Pathogen and Plant Protecting Bacteria (PPPPB) culture collection, ARC-PPRI, Pretoria, South Africa; ICMP accessions – International Collection of Microorganisms from Plants, Auckland, New Zealand; DAR and RAMI accessions – R. Cother, New South Wales Agricultural, Australia; 0198-28 and 0696-21 from D.A Cooksey, Department of Plant Pathology, University of California, USA; SUPP and Yomagi accessions – Y. Takikawa, Laboratory of Plant Pathology, Shizuoka University, Japan; Jan-97, Jan-98, and Jan-98 – R. Gitaitis, College of Agricultural and Environmental Sciences, University of Georgia, USA. Products were amplified by PCR with the primers indicated in Table S1. Gray blocks with a + indicate the presence of bands visualized with agarose electrophoresis for xerC1, xerC2, topB, pilV, traI, ssfD, and mae1 products and the absence of a product for sit1 or sit2. Strains indicated in bold are those for which genome sequences are available*.

PCR amplification was undertaken with a standardized PCR program (94°C for 5 min; 30× {94°C for 1 min, 55°C for 1 min, 72°C for 1 min}; 72°C for 5 min), and the resultant amplicons were visualized, after agarose gel electrophoresis with GelRed™ nucleic acids stain (Biotium, California, USA), using a UV transilluminator. The presence of an insert within tRNA-Phe site 1 or site 2 was confirmed by the absence of a band for Sit1 and/or Sit2, while the presence of an ICE*Pan* element in these sites was determined on the basis of the presence of bands for the *xerC1, xerC2, topB, pilV, and traI* gene products. The presence of a putative antibiotic biosynthetic locus and the *mae1*-containing *umuDC* island was confirmed through the presence of a band for the *ssfD* and *mae1* gene products, respectively.

## Results and discussion

### General properties of the *P. ananatis* integrative conjugative elements

**Figure 1:**
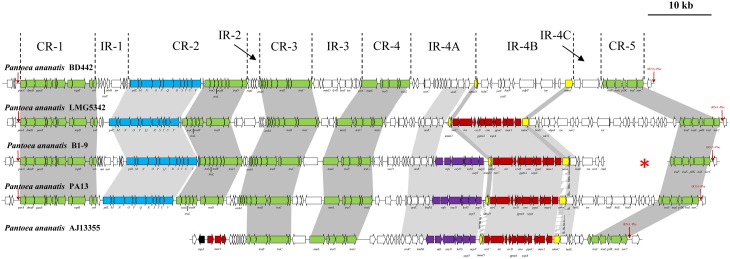
**Schematic diagram of the ***P. ananatis*** integrative conjugative elements ICE***Pan*****. Core regions CR1-5 and island regions (IR1-4) are indicated. Green arrows indicate the conserved genes in the CRs, blue arrows represent the *pilLMNOPQRSTUV* locus, red arrows the conserved IR-4B genes in ICE*Pan*AJ13355, ICE*Pan*B1-9, ICE*Pan*LMG5342 and ICE*Pan*PA13, while the putative antibiotic biosynthetic locus is indicated by purple arrows. The red arrows at the 5′ and 3′ ends indicate the tRNA-Phe integration site for ICE*Pan*, while the transposase flanking the ICE*Pan*AJ13355 is represented by a black arrow. The red star shows the non-contiguous ends of the contigs that make up ICE*Pan*B1-9.

Analysis of eight *P. ananatis* genomes revealed the presence of integrative and conjugative elements integrated on the chromosomes of five strains (Table 1; Figure 1). These elements belong to a novel family, ICE*Pan*, named in accordance with the nomenclatural system proposed by Burrus et al. (2002), to reflect the species, *P. ananatis* in which they were identified, and the strain numbers to distinguish between the different ICE*Pan* elements. The ICE*Pan* elements vary in size from 67.4 (ICE*Pan*AJ13355) to 110 kb (ICE*Pan*LMG5342) and carry between 59 and 113 protein coding sequences (CDSs). The G+C contents of the ICEs (53.2–53.9%) are similar to those of the rest of the chromosome (53.5–53.7% for the complete chromosomes of AJ13355, LMG5342, and PA13). As is the case for the majority of genomic islands, it has been observed that most ICEs are integrated within tRNA genes, which serve as hotspots for recombination (Burrus et al., 2002; Boyd et al., 2009). All five ICE*Pan* elements are integrated into one of two copies of identical phenylalanine-specific tRNAs, *pheU*/*V* (Table 1) present on all the genomes. Similarly, the ICEs CTnscr94 of *Salmonella enterica* subsp. *enterica* serovar Senftenberg 5494-57, YAPI of *Yersinia pseudotuberculosis* 32777 and ICE*Ec2* of *Escherichia coli* BEN374 (Hochhut et al., 1997; Collyn et al., 2002; Roche et al., 2010) are integrated into tRNA-Phe genes. It has been observed that ICE integrases may have evolved to drive ICE insertion within specific tRNA genes. This targeted integration may ensure that essential loci and highly expressed tRNAs, where ICE integration may affect cellular fitness, are avoided (Boyd et al., 2009). While four of the ICE*Pan* elements were integrated in one tRNA-Phe site (Site 1; adjacent to *yjdC* encoding a putative HTH-transcriptional regulator), the ICE*Pan*AJ13355 was integrated within the second identical tRNA-Phe gene copy (Site 2; adjacent to serine phosphatase gene *rbsU*). The ICE-associated integrase (XerC1) of the four ICE*Pan* elements integrated in the first site shared 99.4–100% amino acid identity among them, while the ICE*Pan*AJ13355 integrase (XerC2) shares lower sequence identity (58.5% average amino acid identity) with XerC1. Variability in the integrase sequence may thus dictate the ICE*Pan* integration site.

### The ICE*Pan* elements contain core modules for integration, maintenance, and conjugative transfer

Comparison of the ICE*Pan* elements of the five strains revealed that they comprise of four (ICE*Pan*AJ13355) or five (ICE*Pan*B1-9, ICEPanBD442, ICE*Pan*LMG5342, and ICE*Pan*PA13) conserved syntenic blocks, CR 1–5, with regions containing non-conserved genes, IR 1–4, interspersed between these blocks (Figure 1). The protein complements of the ICE*Pan* CRs were compared to characterized ICEs to identify the three core functional modules within the CR blocks of the ICE*Pan* elements.

The tyrosine recombinase (XerC), which drives both the integration and excision of various ICEs including those of the SXT/R391 family (Boyd et al., 2009), is localized in CR-5 of the ICE*Pan*. A large number of proteins with putative roles in the conjugative transfer of ICE*Pan* are interspersed within the CRs. These include orthologs of the relaxase TraI (CR-5), Type IV coupling factor TraG (CR-4), conjugative transfer ATPase TraC (CR-3) and putative conjugative transfer proteins TraE and TraF (CR-5). Located within CR-2 are genes encoding orthologs of another conjugation coupling factor TraD, exported transglycosylase TraL and putative conjugative transfer protein TraW. CR-2 also includes a ~11 kb locus encoding 11 proteins, PilL-V, sharing 50 and 46.2% average amino acid identity with the Pil proteins of the ICEs CTnscr94 in *S. enterica* (CAX68107.1-CAX68117.1) and YAPI in *Y. pseudotuberculosis* (CAF28485.1-CAF28494.1), respectively (Figure 1). The encoded type IV pilus was initially thought to function as a virulence factor, with deletion of the *Y. pseudotuberculosis pil* locus resulting in decreased pathogenicity in mice which were infected orally (Collyn et al., 2002). However, more recent analysis of *E. coli* ICE*Ec2* has shown that this pilus plays a role in mating pair formation and conjugal transfer, and the decreased pathogenicity of the *Y. pseudotuberculosis pil* mutant may rather result from accessory elements on YAPI (Collyn et al., 2006; Roche et al., 2010). The chromosome partitioning protein ParA/Soj and plasmid maintenance protein ParB have been demonstrated to play a role in the maintenance and vertical transmission of the excised ICE (Wozniak and Waldor, 2009). Genes encoding orthologs of these proteins are localized within ICE*Pan* CR-1. Our analyses thus revealed that the core modules for integration and excision, maintenance and conjugative dissemination are present in the ICE*Pan* regions of four of the five sequenced ICE*Pan*-containing strains, suggesting that they represent functional and transmissible ICEs. The absence of CR-1 and -2, which carry genes for ICE dissemination in the other strains, suggests that ICE*Pan*AJ13355 has lost its functionality as a transmissible element. The absence of CR-1 is puzzling, as this region is involved in the vertical maintenance of the ICE element during cell division and replication. One possibility is that the distinct chromosomal integration site and/or the presence of integrase XerC2 within the ICE*Pan*AJ13355 prevent its excision. In its un-excised form it may then be replicated as part of the chromosome.

### The ICE*Pan* family elements are closely related to other enterobacterial ICEs and share a common origin with conjugative plasmids of *Erwinia amylovora* and *Erwinia billingiae*

**Figure 2:**
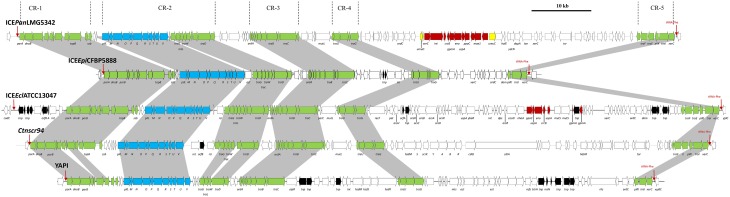
**Schematic diagram of ICE***Pan***LMG5342 and closely related ICEs showing the extensive synteny that exists between the CRs**. ICE*Epi*CFBP5888 and ICE*Ecl*ATCC13047 were identified from the genome sequences of *E. piriflorinigrans* CFBP 5888^T^ and *E. cloacae* subsp. *cloacae* ATCC13047^T^, respectively. The Genbank files for CTnscr94 of *S. enterica* subsp. *enterica* serovar Senftenberg 5494-57 and YAPI of *Y. pseudotuberculosis* 32777 were obtained from the ICEberg database (Bi et al., 2012). Dark gray shaded regions and green arrows indicate the conserved CRs between the ICEs. Black arrows represent transposases.

**Figure 3:**
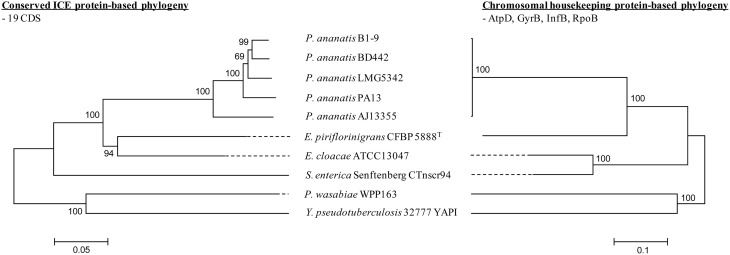
**Phylogeny of ICE***Pan*** and related ICEs and comparison to chromosomal house-keeping marker phylogeny**. Alignments were done with MAFFT (Katoh and Standley, 2013) using the concatenated amino acid sequences of the ICE elements (19 conserved CDSs) and house-keeping markers (AtpD, GyrB, InfB, and RpoB). Phylogenies were constructed using MEGA v 5.2 (Tamura et al., 2011), using the Neighbor-joining algorithm with complete gap deletion, Poisson correction and bootstrap analysis (*n* = 1000).

Nineteen CDSs conserved in all five ICE*Pan* elements showed significant sequence identity to proteins encoded on various characterized enterobacterial ICEs, including Ctnscr94 of *S. enterica* subsp. *enterica* serovar Senftenberg 5494-57 and YAPI of *Y. pseudotuberculosis* 32777 (Table 3). The conserved ICE*Pan* CDSs showed highest sequence similarity to those encoded on uncharacterized ICEs identified in the genomes of *Enterobacter cloacae* ATCC13047 (ICE*Ecl*ATCC13047) and *Erwinia piriflorinigrans* CFBP5888^T^ (ICE*Epi*CFBP5888) (Table 3) (Ren et al., 2010; Smits et al., 2013). Alignment of the ICE loci (Figure 2) also indicated a high level of synteny between ICE*Pan* and the enterobacterial ICEs. A phylogeny based on the concatenated protein products of the conserved ICE CDSs showed similar clustering to a phylogeny constructed on the basis of the concatenated amino acid sequences of four chromosomal house-keeping markers (Figure 3). This suggests that ICE*Pan* and the compared enterobacterial ICEs were derived from a common ancestor, and have undergone subsequent divergence in parallel with the bacterial chromosome.

**Table 3:** **Average amino acid identities between ICE***Pan*** and related ICEs**.

	**AJ13355**	**B1-9**	**BD442**	**LMG5342**	**PA13**	**ECL**	**EPIR**	**CtnScr94**	**YAPI**	**Pwa**
ICE*Pan*AJ13355	−	90.0	90.3	89.4	89.1	75.4	74.6	65.9	63.1	62.7
ICE*Pan*B1−9		−	96.0	96.0	94.1	75.2	72.3	64.6	62.6	62.2
ICE*Pan*BD442			−	95.2	93.4	75.3	72.7	64.6	62.4	61.7
ICE*Pan*LMG5342				−	94.9	75.5	72.4	64.8	62.4	61.9
ICE*Pan*PA13					−	74.4	71.6	64.7	62.0	61.6
ICE*Ecl*ATCC13047						−	78.9	67.7	64.9	64.3
ICE*Epi*CFBP5888							−	67.5	64.0	62.7
CTnscr94								−	60.6	61.2
YAPI									−	69.5
ICE*Pwa*WPP163										−

*The average amino acid identities (%) between pair-wise compared translated ICE CDS sets was calculated on the basis of 16 conserved CDSs, where the sum of identities for each CDS was divided by the sum total of the alignment lengths*.

**Figure 4:**
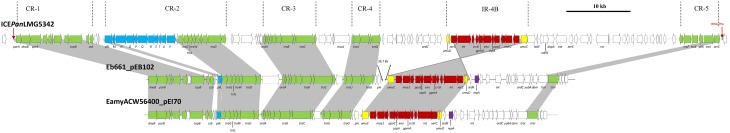
**Schematic diagram of ICE***Pan***LMG5342 and the phylogenetically related conjugative plasmids pEI70 of ***E. amylovora*** ACW56400 and pEb102 of ***E. billingiae*** Eb661**. ICE*Pan*LMG5342 is aligned with the plasmids pEI70 of *E. amylovora* ATCC56400 (NC_018999.1) and pEb102 of *E. billingiae* Eb661 (NC_014304.1). Dark gray shaded regions and green arrows indicate the conserved CRs between the ICEs. The conserved umuCD island is marked by red arrows, with the umuC fragments and umuD genes colored in yellow, and the plasmid replication gene repA in pEI70 and pEb102 is colored purple.

BlastP analyses of the translated ICE*Pan* CDSs against the NCBI non-redundant (nr) protein database revealed a number of orthologous CDSs occurring in the conjugative plasmids of *E. amylovora* ACW56400 (pEI70 – NC_018999.1; ~65 kb) and *E. billingiae* Eb661 (pEB102 – NC_014304.1; ~102 kb) (Figure 4) (Kube et al., 2010; Llop et al., 2011). Between 36 and 54 of the 114 proteins encoded on pEB102 (60.3% average amino acid identity) and 35–53 of the 70 proteins encoded on pEI70 (60.1% average amino acid identity) shared orthology with proteins encoded on the ICEPan elements. The lower number of orthologous CDSs shared between pEI70/pEb102 and ICE*Pan*AJ13355 is due to the absence of CR-1 and -2 from the latter element. Among the conserved plasmid/ICE CDSs are those in the maintenance (ParAB) and conjugative transfer (TraDEFGILW) modules. With the exception of a shared PilL ortholog, no other orthologs of proteins encoded in the ICE*Pan pil* locus are present in pEb102 or pEI70, suggesting that Pil pilus-associated conjugative transfer is specific to ICE elements. Also absent from the conjugative plasmids is an ortholog of the integration and excision module protein XerC indicative of pEI70 and pEb102 being maintained as plasmids rather than being chromosomally integrated. The presence of a number of orthologous CDSs within the ICE*Pan* loci and the plasmid pEI70 and pEb102 and the presence of these orthologs in syntenous blocks suggest a common origin for the ICE*Pan* family elements and the *Erwinia* plasmids (Figure 4). A phylogeny constructed on the basis of the amino acid sequences of 21 CDSs conserved in pEI70, pEb102, all five ICE*Pan* elements, ICE*Ecl*ATCC13047 and ICE*Epi*CFBP5888 showed that ICE*Pan* is more closely related to the enterobacterial ICE elements than pEb102 and pEI70 (Supplementary Figure S1). This is incongruent with the phylogeny on the basis of the house-keeping markers, which thus suggests that the common ancestor of the ICE/plasmids pre-dates species divergence.

### ICE*Pan* elements are prevalent among *P. ananatis* strains isolated from different sources

The prevalence of ICEs in *P. ananatis* was determined through PCR amplification with primers designed on the basis of conserved ICE genes and integration sites (Table S1). Initially, two sets of primers *sit1F/R* and *sit2F/R* were designed to cover the two genomic tRNA-Phe regions on the basis of conserved flanking sequences in strains which did not have an insertion at these sites. No amplicons were observed in 31 out of 46 strains (67.4% of strains tested), indicating that a large insert is integrated at one or both of the tRNA-Phe sites. Of these, 11 insertions occur in the tRNA-Phe site occupied by ICE*Pan*B1-9, ICE*Pan*BD442, ICE*Pan*LMG5342 and ICE*Pan*PA13 (*sit1*), nine solely in the tRNA-Phe site of ICE*Pan*AJ13355 (*sit2*) while 11 strains had large inserts integrated at both sites (Table 2). This would suggest that in the latter strains, a third functional copy of the tRNA-Phe gene exists, or that the tRNA-Phe gene(s) is located at a different site. As these primers only give an indication of the presence of an insertion at a given tRNA-Phe site, further primers were designed on the basis of conserved genes in the ICE*Pan* elements.

PCR amplification with primers designed on the conserved sequences of the ICE*Pan*B1-9, ICE*Pan*BD442, ICE*Pan*LMG5342, and ICE*Pan*PA13 integrase (*xerC1F/R*) produced amplicons in six additional strains, while those based on the ICE*Pan*AJ13355 integrase (*xerC2F/R*) amplified products in six more strains. Further primers designed on the basis of the CR genes coding for topisomerase B (*topB*), type IV pilus shufflon (*pilV*) and relaxase/helicase (*traI*) amplified products in 22, 23, and 20 strains, respectively (Table 2). There was some variability in the presence of the conserved ICE genes among the strains. For example, nine strains were positive for one or more of the *traI, pilV, topB* gene fragments, while they were negative for both the XerC1 and XerC2 integrase primer sets. Similarly, while ICE*Pan*AJ13355 was missing the core regions containing the *topB* (CR-1) and *pilV* (CR-2) genes, products were obtained for either or both of the gene fragments in six strains which were positive for the ICE*Pan*AJ13355 integrase XerC2. In addition to the variability observed among ICE*Pan* elements of the sequenced strains, there thus appears to be further variability among the ICE elements of other *P. ananatis* strains. Variability in the conserved sequences, as was observed for the distinct integrases, may also result in no amplification occurring for conserved ICE genes among *P. ananatis* strains. Nevertheless, 24 out of 46 (52% of tested strains) *P. ananatis* strains were positive for two or more of the conserved ICE genes tested (Table 2), which suggests that integrative conjugative elements are relatively common among strains of the species.

### ICE*Pan* elements carry extensive non-conserved cargo genes with a potential role in fitness, stress response, and antibiosis

The ICE*Pan* elements of all five strains contain four large non-conserved cargo regions, IR 1–4 (Figure 1). IR4 has further been subdivided into IR-4A and IR-4C, separated by a region flanked by the *umuD* and partial *umu*C genes, IR-4B, which is discussed in further detail below. The IRs contribute between 45.7% (ICE*Pan*BD442) and 65.7% (ICE*Pan*AJ13355) of the total ICE*Pan* size (Table 4). The G+C contents of the ICE cargo and core regions were determined. This showed that while the G+C contents of ICE*Pan* (average G+C content: 53.3%) is similar to that of the chromosomes (53.7%), the G+C content of the core regions (56.9%) is somewhat higher and that of the IRs is lower (50.0%) than that of the chromosome (Table 4). This suggests that the current ICE*Pan* structures may have arisen through distinct horizontal acquisition events of the core and island regions. Between 44 (ICE*Pan*AJ13355) and 67 (ICE*Pan*LMG5342) CDSs are encoded in IR1-4. Our previous pan-genome comparison of eight *P. ananatis* strains revealed a sizeable accessory genome (1690 CDS—30.4% of the pan-genome) for the species (De Maayer et al., 2014). A total of 124 distinct CDSs are encoded in the IRs of the ICE*Pan* elements, suggesting this genomic element contributes substantially to the accessory fraction of the pan-genome and that ICE*Pan* plays a major role in the diversification of *P. ananatis*.

**Table 4:** **Characteristics of the ICEPan and its core (CR) and island (IR) regions**.

	**ICE**	**CR**	**IR**	**CR**	**IR**	**ICE**	**CR**	**IR**	**Genome**	**ICE**	**CR**	**IR**
	**Size (kb)**	**Size (nt)**	**Size (nt)**	**% Total**	**% Total**	**G+C (%)**	**G+C (%)**	**G+C (%)**	**G+C (%)**	**# CDSs**	**# CDSs**	**# CDSs**
ICE*Pan*AJ13355	59.4	20.4	39.0	34.32	65.68	52.67	56.76	50.53	53.76	67	23	44
ICE*Pan*B1-9	99.2	52.7	46.5	53.11	46.89	53.7	56.87	50.12	53.62	111	47	64
ICE*Pan*BD442	98.8	53.7	45.2	54.3	45.7	53.22	56.88	48.87	53.78	109	47	62
ICE*Pan*LMG5342	110.3	51.9	58.4	47.05	52.95	53.22	56.96	49.89	53.45	114	47	67
ICE*Pan*PA13	107.2	53.5	53.7	49.91	50.09	53.84	57.02	50.67	53.66	111	47	64

*The sizes (number of nucleotides) and proportions (%) of the CRs and IRs in relation to the total ICEPan element, G+C contents (%), number and proportions (%) of CDSs on the ICE, CRs, and IRs are shown. The average G+C content of the genomes are given, with a indicating those strains for which the genomic G+C contents were determined on the basis of available contigs for draft genomes*.

Several genes within the ICE*Pan* IRs encode orthologs of characterized stress response mechanisms (Table S2). One means of stress response utilized by Gram-negative bacteria is through the production and activation of alternative RNA polymerase σ factors. These regulate the transcription of a large number of different genes that allow the cell to tolerate or counteract various stresses including starvation, osmotic stress, and oxidative and DNA damage (Bougdour and Gottesman, 2007). It is imperative that σ factor concentrations, activity and stability are tightly regulated. Several σ factor stabilizing proteins have been identified including the ATP-dependent protein ClpXP and the anti-adaptor protein IraP which stabilizes σ factor during phosphate starvation (Bougdour and Gottesman, 2007). Genes within the IR-4A of ICE*Pan*PA13 and ICE*Pan*BD442 encode orthologs of ClpXP, while an IraP ortholog is encoded in IR-1 of ICE*Pan*BD442. Another means of stress response is through the expression of Universal Stress Proteins (Usp). UspA of *E. coli* is produced in response to starvation, osmotic and heat shock, as well as exposure to heavy metals and antimicrobial agents (Kvint et al., 2003). Orthologs of UspA are encoded within the IR-4B of all ICE*Pan* elements with the exception of ICE*Pan*BD442. Bacteria associated with plants are frequently exposed to reactive oxygen species such as superoxide anions and hydroperoxides which form an integral part of plant defense responses. A gene localized within the IR-1 of ICE*Pan*BD442 encodes an ortholog of OhrR, a sensor and regulator of organic hydroperoxide resistance (Panmanee et al., 2006). Within IR-4B of this same strain a gene encoding an ortholog of the σ^S^-regulated manganese-catalase KatN, which has been demonstrated to degrade hydroperoxides into water and oxygen in *S. enterica*, is present (Robbe-Saule et al., 2001). As is the case for the *katN* gene in *S. enterica*, the ICE*Pan*BD442 gene is localized adjacent to genes showing extensive sequence identity to *yciEFG*, which are likewise thought to play a role in oxidative stress response (Hindupur et al., 2006). Upstream of the ICE*Pan*BD442 *katN* gene, a gene encoding an ortholog of the oxidoreductase YdeP involved in acid resistance in *Shigella flexneri* (Oglesby et al., 2005), is present.

Aside from roles in stress response, several ICE*Pan* CDSs may contribute to the fitness of the ICE carrying strains. A locus within IR-4C of ICE*Pan*PA13 (Pagr_3794-Pagr_3798) encodes five proteins sharing 61.8% average amino acid identity with a predicted iron transport system in *Agrobacterium tumefaciens* 5A (AT5A_20056.1-AT5A_20076.1). This locus may allow *P. ananatis* PA13 to compete for this limiting nutrient within the plant host. Also interspersed among the IR-1, -4, and -5 of all ICE*Pan* elements with the exception of ICE*Pan*AJ13355 are six genes encoding five distinct methyl-accepting chemotaxis proteins (MCPs). MCPs sense the presence of certain attractants and repellents to determine the direction of bacterial motility, toward or away from these chemical signals (Baker et al., 2006). ICE*Pan* elements may thus play a role in the spread of MCPs for the response to different chemical stimuli, including those they may encounter in novel environmental niches.

Analyses of the ICEs of several microorganisms have shown the presence of loci for the biosynthesis of secondary metabolites such as siderophores and antibiotics on these elements (Ghinet et al., 2011). The ICE*Pan* sequences were compared against the antiSMASH (antibiotics and secondary metabolite analysis shell) server (Blin et al., 2013). A ~7.8 kb locus encoding seven proteins involved in the biosynthesis of a predicted secondary metabolite is present in the IR-4A regions of ICE*Pan*B1-9, ICE*Pan*PA13 and ICE*Pan*AJ13355 (Figure 1; Table S2). The encoded proteins share 99.3–99.9% average amino acid identity among the three strains. Two proteins encode orthologs of the fosmomycin biosynthetic proteins Fom1 and Fom2 of *Streptomyces fradiae* (ACG70831.1-ACG70832.1—37.6% average amino acid identity). Fom1 encodes a phosphoenolpyruvate (PEP) mutase which catalyzes the first step of fosmomycin biosynthesis, converting PEP into phosphonopyruvate (PnPy), which is consequently converted by PnPy decarboxylase (Fom2) into phosphonoacetaldehyde (Woodyer et al., 2006). Two further proteins share 48.3% average amino acid identity to amidotransferase SsfD (ADE35421.1) and acyl carrier protein SsfC (ADE35420.1) of *Streptomyces* sp. SF2575 which are required for the production of the malonamate starter unit of tetracyclines (Pickens et al., 2010). Another protein shows weak sequence identity (34.5% average amino acid identity) to StrU of *Streptomyces griseus* (CAH94303.1), a dehydrogenase involved in the final processing of streptomycin. Also encoded in ICE*Pan* IR-4A of these three strains is an ortholog of the AfsA protein of *Streptomyces virginiae* MAF10-06014 (BAA23611.1). AfsA is a diffusible γ-butyrolactone autoregulator which regulates the production of secondary metabolites in *Streptomyces* spp. (Healy et al., 2009). The presence of this locus in the ICE*Pan* IR-4A region suggests that *P. ananatis* AJ13355, PA13 and B1-9 are capable of producing a secondary metabolite with a potential role in antibiosis. Amplification with primers designed on the basis of the *ssfD* nucleotide sequences showed that two additional rice-pathogenic strains from Australia and one *Eucalyptus*-pathogenic strain from South Africa, may potentially encode this putative secondary metabolite biosynthetic locus (Table 2). Within IR-1 of ICE*Pan*B1-9 are two genes encoding proteins with weak sequence identity (31.3% average amino acid identity) to the alveicin bacteriocin A Aat (CDD cl04134: microcin superfamily) of *Hafnia alvei* and its cognate immunity protein Aai (CDD pfam01024—colicin pore forming domain). Bacteriocins are bacterial toxins that kill closely related bacteria, while the immunity protein provides self-protection against the toxin (Wertz and Riley, 2004). The presence of bacteriocin and putative antibiotic biosynthetic loci within the IR regions of the ICE*Pan* family elements suggest this element may confer a potential competitive advantage over other bacteria and fungi sharing the same environmental niche and thus contribute to the fitness of ICE*Pan*-positive *P. ananatis* strains.

### A non-conserved island is integrated into the *UmuDC* locus

**Figure 5:**
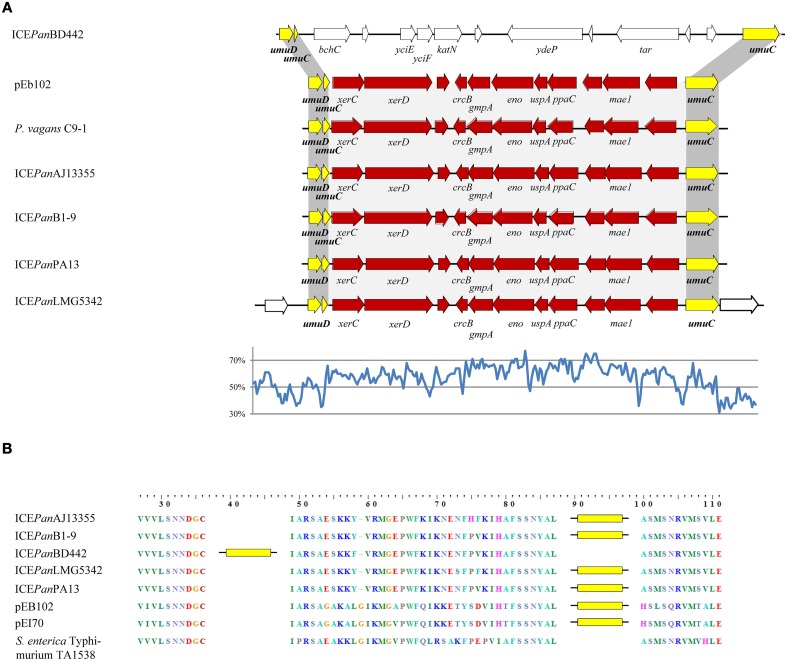
**Schematic diagram of the ***umuDC*** island in ICE***Pan*** and related elements (A) and the integration site within the ***umuC*** amino acid sequence (B). (A)** The ICE*Pan umuDC* islands are aligned with the *umuDC* island from the conjugative plasmids of *E. amylovora* ACW56400 (pEI70) and *E. billingiae* Eb661 (pEb102) and the chromosomally integrated *umuDC* island of *P. vagans* C9-1. A line graph of the G+C contents (%) for ICE*Pan*LMG5342, determined on the basis of G+C contents for 100 nt steps with 50 nt overlap, is shown. **(B)** The amino acid sequences of the ICE*Pan* UmuC protein were aligned with that of CTnscr94, and the insertions are indicated by yellow bars.

Localized within IR-4 of the ICE*Pan* of all five strains are genes encoding proteins with extensive sequence identity to the SOS damage response proteins UmuC and UmuD. In response to DNA damage, as a result of genotoxic stresses such as UV irradiation or exposure to mitomycin C, these two proteins effect error-prone replication across the damaged DNA lesion, also known as SOS mutagenesis (Hare et al., 2006). The presence of UmuDC orthologs further supports a role for ICE*Pan* in stress response. BlastN analysis of the ICE*Pan* IR-4B CDS sets revealed that the *umuC* gene is disrupted by a non-conserved insertion (Figure 5A). A similar disruption of the *umuC* gene could be observed on the chromosome of *Pantoea vagans* C9-1 and the *Erwinia* plasmids pEb102 and pEI70. This insertion is ~11 kb in size in ICE*Pan*B1-9, ICE*Pan*AJ13355, ICE*Pan*LMG5342, and ICE*Pan*PA13 with an average G+C content of 60.3%, substantially higher than the average G+C% for the ICE*Pan* element (53.3%) and the chromosome (Figure 5A). By contrast the *umuC* insertion in ICE*Pan*BD442 is ~13.7 kb in size with a G+C content of 49.0%. The amino acid sequences of the disrupted *umuC* genes were aligned (Figure 5B). This showed that the integron is inserted at amino acid position 88 in the UmuC protein of the *Erwinia* plasmids pEB102, pEI70 and all ICE*Pan* with the exception of ICE*Pan*BD442. In the latter, the insertion is integrated in UmuC after position 37.

The ICE*Pan*BD442 insertion encodes 11 proteins not encoded in the other ICE*Pan*, while 11 CDSs are conserved among the other four ICE*Pan* (99.2% average amino acid identity) (Table S2). The ICE*Pan*BD442 *umuDC* island CDSs include the catalase KatN, YciEFG and YdeP orthologs, which may play a role in oxidative and acid stress response as discussed above, as well as a methyl-accepting chemotaxis protein (Figure 5A). Proteins encoded within ICE*Pan*AJ13355, ICE*Pan*B1-9, ICE*Pan*LMG5342, and ICE*Pan*PA13 *umuDC* region include an ortholog of the Mae1 protein in the yeast *Schizosaccharomyces pombe* which plays a role in the uptake of the dicarboxylic acid substrates L-malate, succinate, and malonic acid that are central to the tricarboxylic acid (TCA) pathway (Grobler et al., 1995). Also encoded within this island are orthologs of the 2,3-bisphosphoglycerate-independent phosphoglyceratemutase GpmA and enolase Eno which catalyze the reversible conversion of 3-phospho-D-glycerate to PEP, involved in the glycolysis and gluconeogenesis (Nurmohamed et al., 2010). The *umuDC* island-encoded enzymes thus play a likely role in energy production and conversion. Also included in the region is a gene (*ppaC*) encoding a manganese-dependent inorganic pyrophosphatase, which removes pyrophosphate generated as a by-product of many biosynthetic and metabolic reactions, as well as a gene encoding the universal stress protein UspA, which is discussed above. Finally, orthologs of the recombinases XerC and XerD are encoded adjacent to the small 5′ fragment of *umuC*.

The 11 translated CDSs in the ICE*Pan* elements of AJ13355, B1-9, LMG5342, and PA13 share 99.3% average amino acid identity, similar to the average amino acid identity for the core genome CDS sets (99.3%) (De Maayer et al., 2014). By contrast the IR-4B CDSs share much higher average amino acid identity (~92.0%) with orthologs in *P. vagans* C9-1, *E. billingiae* Eb661, and *E. amylovora* ACW56400 than the genomic average (84.8, 76.0, and 76.3%, respectively) (Table S3). Most striking is the comparison of the 11 *umuC*-inserted CDSs on the chromosome of *P. vagans* C9-1 and those encoded on pEb102 and pEI70, which share an average amino acid identity of 99.7 and 100%, respectively (Table S3), which is substantially higher than the genomic average amino acid identities (76.4, 77.2, and 81.0% average amino acid identity for *P. vagans* C9-1 vs. *E. billingiae* Eb661 and *E. amylovora* ACW56400, and Eb661 vs. ACW56400, respectively). Alignment of the nucleotide sequences for these regions shows only three nucleotide polymorphisms between the *umuDC* islands of *P. vagans* C9-1 and *E. amylovora* pEI70, while those of C9-1 and *E. billingiae* Eb661 share 100% nucleotide identity. This suggests very recent inter-genera horizontal exchange of the *umuDC* island regions between the plasmids of *E. amylovora* ACW56400 and *E. billingiae* Eb661, ICE*Pan*AJ13355, ICE*Pan*B1-9, ICE*Pan*LMG5342, and ICE*Pan*PA13 and the chromosome of *P. vagans* C9-1 (Supplementary Figure S2).

While the functions encoded in the *umuDC* islands of the ICE*Pan, P. vagans* C9-1 chromosome and *Erwinia* plasmids, remain to be elucidated, we hypothesize two potential reasons for the integration of this island into the *umuC* gene. Firstly, as the *umuDC* is transcriptionally upregulated in response to stress, the insertion into the *umuC* reading frame may result in the genes on the island being expressed. This hypothesis is supported by observations in *Acinetobacter baylyi* ADP1, where a truncated *umuC* is also present and *umuDC* regulates the expression of DNA-damage inducible gene *ddrR*, which is located upstream of *umuD* (Hare et al., 2006). The truncated *umuC* may thus serve as a DNA-damage inducible regulator for genes on the island which may potentially play a role in stress response. Alternatively, the recombinases encoded by the *xerC* and *xerD* genes located directly upstream of the truncated 5′ region of *umuC* (Figure 5A) may function in a similar fashion to the integration and excision core module tyrosine recombinase XerC in ICE*Pan* CR-5. The stress-induced upregulation of *umuC* may thus drive the excision and dissemination of the island and its encoded genes. A BlastP analysis with the UmuC and UmuD protein sequences against the ICEberg dataset showed that orthologs were present in 21 out of 270 complete ICE elements. Of these, an island insertion was observed in the *umuC* gene of 10 ICE elements, integrated mostly in ICEs of *Vibrio* spp., but also one *Pseudomonas aeruginosa* and one *Streptococcus pyogenes* ICE (Table S4). The islands inserted into the *umuC* gene in the ICE elements of these microorganisms carried genes implicated in mercury resistance and resistance to several antibiotics including tetracycline, chloramphenicol/florfenicol and streptomycin (Beaber et al., 2002; Battle et al., 2008). In the context of the above hypotheses, the *umuC* island may thus play a major role in the regulation and/or dissemination of important phenotypes from both a clinical and ecological perspective.

## Conclusions

ICEs of the novel ICE*Pan* family are a common feature among *P. ananatis* strains isolated from various environmental sources and hosts, and with different lifestyles. *In silico* characterization of the ICE*Pan* elements showed similar ICEs are found in other enterobacteria and that these ICEs, and conjugative plasmids in *E. billingiae* and *E. amylovora*, are likely derived from a common ancestor. ICE*Pan* contain the core modules required for their chromosomal integration, maintenance and dissemination. The presence of these core elements, however, does not provide definitive proof that the ICE*Pan* elements can excise, circularize and integrate within the chromosome. Currently, assays are being performed to determine whether ICE*Pan* represent functional integrative and conjugative elements, particularly ICE*Pan*AJ13355, which lacks two of the core modules. Analyses of the ICE cargo genes suggest a likely role in response to various stresses they may encounter in the environment, including oxidative, pH, and genotoxic stresses. ICE*Pan* elements could therefore play a role in facilitating *P. ananatis* survival under these stresses and provide a potential mechanism for this species to exploit novel ecological niches. With recent interest in the use of *P. ananatis* as biological control agent against phytopathogenic bacteria and fungi, the ICE*Pan* elements, and the putative antibiotic biosynthetic loci encoded on these elements, provide potential targets for the exploration of *P. ananatis* antibiosis phenotypes. Perhaps the most pertinent finding in the ICE*Pan* elements is the integration of an island in the *umuDC* locus, which shows evidence of recent horizontal acquisition between different *Pantoea* species and related genera. Here, we postulate on a potential role of *umuDC* in the dissemination and/or expression of phenotypes encoded on this island. Islands integrated into this locus are furthermore found in other bacterial taxa and encode phenotypes such as antibiotic and heavy metal resistance. It can be reasonably expected that similar features will be identified in the ICEs of many other bacterial taxa whose genomes are sequenced. Considering the clinical and ecological relevance of the phenotypes encoded in *umuDC*, this phenomenon warrants further attention.

## Author contributions

PM, SV, BD, TS, and TC conceived the study. PM, WC, DM, JB, and TS performed experiments and analyses, PM, SV, BD, DC, TS, and TC wrote the original manuscript. All authors contributed to and approved of the final version.

### Conflict of interest statement

The authors declare that the research was conducted in the absence of any commercial or financial relationships that could be construed as a potential conflict of interest.
