# Supplementary material for: Integrative conjugative elements of the ICEPan family play a potential role in Pantoea ananatis ecological diversification and antibiosis
Source: Front Microbiol. 2015 Jun 8;6:576. doi: 10.3389/fmicb.2015.00576 (PMC4458695; doi:10.3389/fmicb.2015.00576)
Supplement: Supplementary file 1 [file Presentation1.PPTX]

## Slide 1
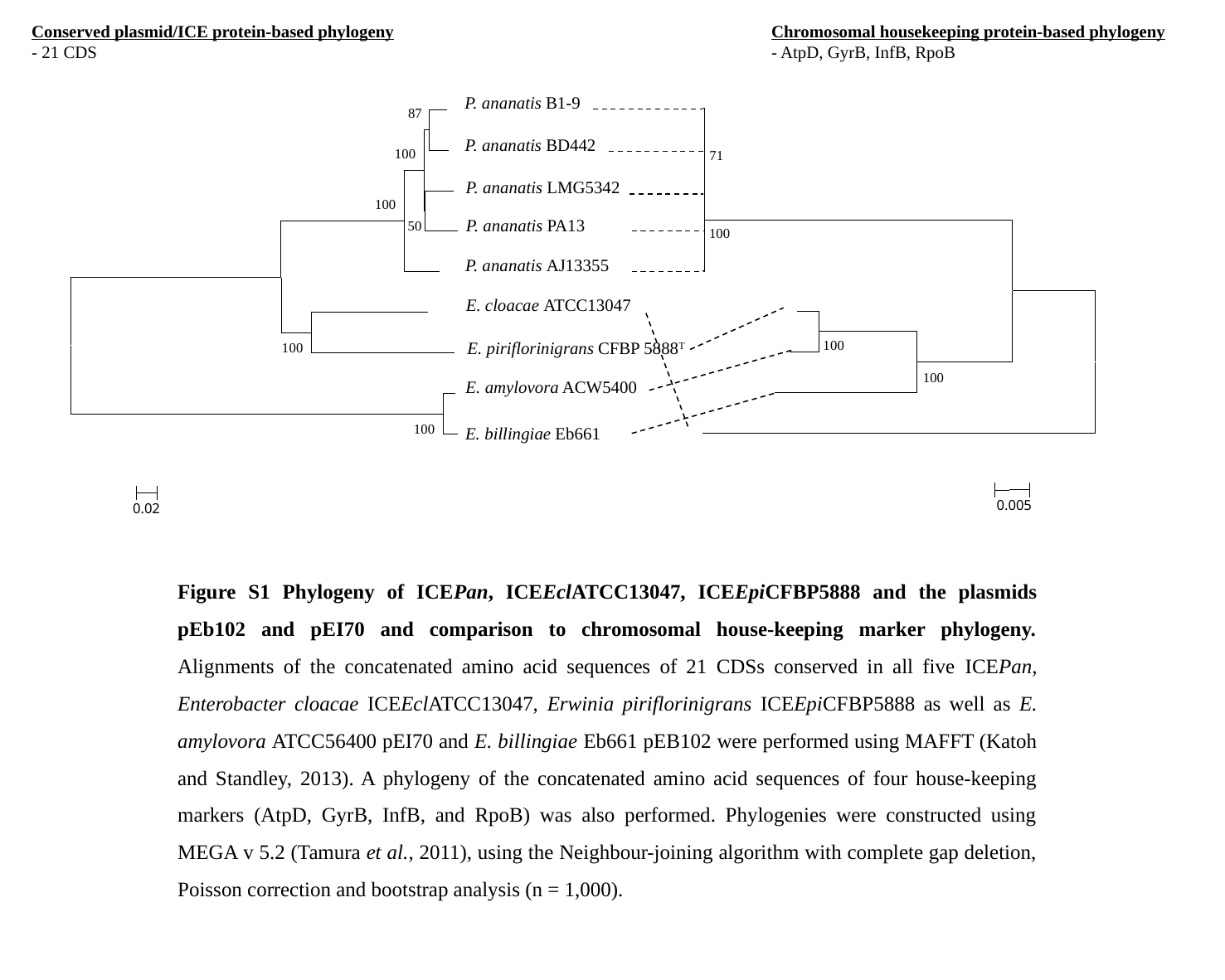

Conserved plasmid/ICE protein-based phylogeny
- 21 CDS
Chromosomal housekeeping protein-based phylogeny
- AtpD, GyrB, InfB, RpoB
0.005
87
100
100
50
100
100
0.02
P. ananatis B1-9
P. ananatis BD442
71
P. ananatis LMG5342
P. ananatis PA13
100
P. ananatis AJ13355
E. cloacae ATCC13047
E. piriflorinigrans CFBP 5888T
100
100
E. amylovora ACW5400
E. billingiae Eb661
Figure S1 Phylogeny of ICEPan, ICEEclATCC13047, ICEEpiCFBP5888 and the plasmids pEb102 and pEI70 and comparison to chromosomal house-keeping marker phylogeny. Alignments of the concatenated amino acid sequences of 21 CDSs conserved in all five ICEPan, Enterobacter cloacae ICEEclATCC13047, Erwinia piriflorinigrans ICEEpiCFBP5888 as well as E. amylovora ATCC56400 pEI70 and E. billingiae Eb661 pEB102 were performed using MAFFT (Katoh and Standley, 2013). A phylogeny of the concatenated amino acid sequences of four house-keeping markers (AtpD, GyrB, InfB, and RpoB) was also performed. Phylogenies were constructed using MEGA v 5.2 (Tamura et al., 2011), using the Neighbour-joining algorithm with complete gap deletion, Poisson correction and bootstrap analysis (n = 1,000).

## Slide 2
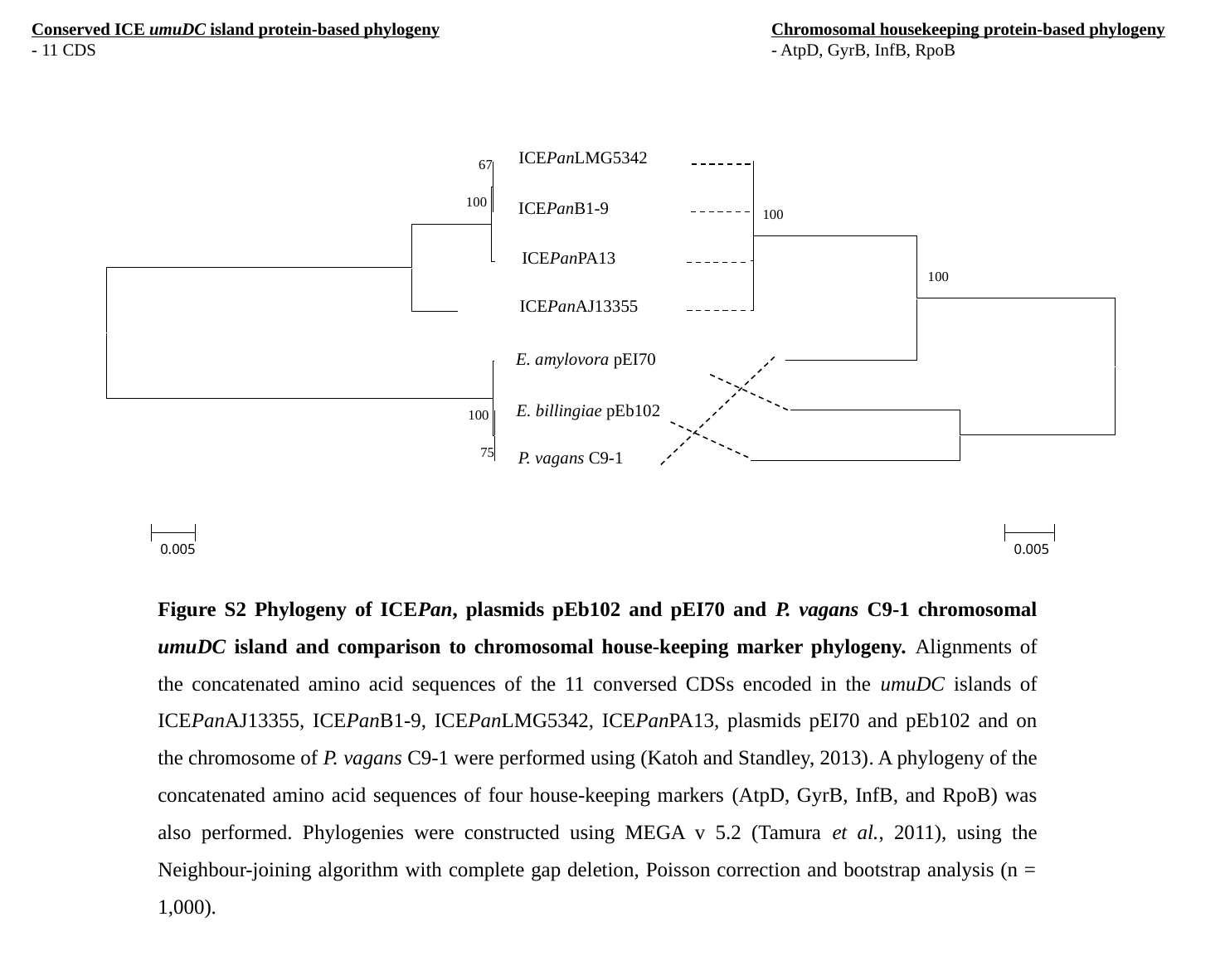

Conserved ICE umuDC island protein-based phylogeny
- 11 CDS
Chromosomal housekeeping protein-based phylogeny
- AtpD, GyrB, InfB, RpoB
100
100
0.005
ICEPanLMG5342
67
ICEPanB1-9
100
ICEPanPA13
ICEPanAJ13355
E. amylovora pEI70
E. billingiae pEb102
100
P. vagans C9-1
75
0.005
Figure S2 Phylogeny of ICEPan, plasmids pEb102 and pEI70 and P. vagans C9-1 chromosomal umuDC island and comparison to chromosomal house-keeping marker phylogeny. Alignments of the concatenated amino acid sequences of the 11 conversed CDSs encoded in the umuDC islands of ICEPanAJ13355, ICEPanB1-9, ICEPanLMG5342, ICEPanPA13, plasmids pEI70 and pEb102 and on the chromosome of P. vagans C9-1 were performed using (Katoh and Standley, 2013). A phylogeny of the concatenated amino acid sequences of four house-keeping markers (AtpD, GyrB, InfB, and RpoB) was also performed. Phylogenies were constructed using MEGA v 5.2 (Tamura et al., 2011), using the Neighbour-joining algorithm with complete gap deletion, Poisson correction and bootstrap analysis (n = 1,000).
